# Obesity and HER 2 overexpression: a common factor for poor prognosis of breast cancer

**DOI:** 10.1186/1477-7800-5-2

**Published:** 2008-02-24

**Authors:** Chaminda Sellahewa, Peter Nightingale, Amtul R Carmichael

**Affiliations:** 1 Department of Surgery, Russells Hall Hospital, Dudley, West Midlands, DY1 2HQ, UK; 2 Wellcome Trust Clinical Research Facility, Birmingham, West Midlands, UK

## Abstract

**Background:**

Both obesity and over-expression of HER II are associated with poor prognosis of breast cancer. In vitro experiments suggest that anti-tumour activity of the anti-obesity drug Orlistat is likely to be due to transcriptional suppression of HER II expression. The overexpression of HER II is also positively correlated with other markers of prognosis of breast cancer such as cathepsin expression.

**Hypothesis:**

The hypothesis we tested was that the obese women with breast cancer might over-express HER II more often than their lean counterparts to account for the poor prognosis.

**Patients and methods:**

One hundred consecutive patients were included in this study. Their body mass indexes were correlated with overexpression of HER II.

**Results:**

There was also no association between oestrogen or progesterone receptor positivity and obesity or HER II over expression in premenopausal or post-menopausal women with breast cancer.

**Conclusion:**

The present study demonstrated that the poor outcome of breast cancer in obese patients is not due to over expression of HER II.

## Introduction

Obesity leads to poor prognosis in women diagnosed with breast cancer even in early stage disease as shown in systematic literature reviews, several large-scale cohort studies and critical reviews such as the Nurse's Health study and cancer prevention study II [1-9]. The women with breast cancer in the highest quartile of BMI are 2.5 times as likely to die of their disease within 5 years of diagnosis compared with women in the lowest quartile of BMI [10].

Recent in vitro experiments suggest that anti-obesity agents Orlistat may have anti-tumour activity which is likely to be due to transcriptional suppression of HER II expression [11]. Orlistat, has potent anti-proliferate and apoptotic effects on breast cancer cells through its ability to block the lipogenic activity of Fatty Acid Synthetase [12]. In vitro experiments also suggest that essential fatty acids such as omega-3 suppress overexpression of HER II at the transcriptional level, which, in turn, interacts synergistically with anti-HER II agents such as Traztuzumab [13]. Traztuzumab is a humanized monoclonal antibody directed against the extracellular domain of the tyrosine kinase receptor HER II. Abnormalities of fatty acid metabolism in the development of breast cancer are well documented and the association between Fatty acid metabolism and HER II antagonists is being discovered REF [14]. Therefore, Obesity may be the missing link between the development of breast cancer and over-expression of HER II because of its effect on essential fatty acids These findings suggest a possible association between obesity, abnormal lipid metabolism and an overexpression of HER II.

Over-expression of HER II is also associated with a poor prognosis of breast cancer as HER II over-expression playS an important role in the development, aggressive behaviour of breast cancer. Overexpression of HER II has been associated with other markers of poor prognosis of breast cancer such as cathepsin D [15].

The hypothesis we tested was that the obese women with breast cancer over-express HER II more often than their lean counterparts to account for the poor prognosis in obese women.

## Patients and methods

One hundred consecutive patients were included in this study. Their height, weight and body mass index were correlated with overexpression of HER II.

### Laboratory methods

The criteria of HER II over expression were used as per Pan Birmingham Protocol. The HER II over expression was measured by immunohistochemistry (DAKO)^®^. The borderline cases were tested with fluorescence in situ hybridisation (Abbott Path Vision)^®^.

### Statistical methods

Using the chi(2)-test, relationships were determined between marker labelling and histological type of cancer, tumour grade, tumour size, axillary lymph node status and age of patient. A p value below 0.05 was considered significant.

## Results

**Figure 1:**
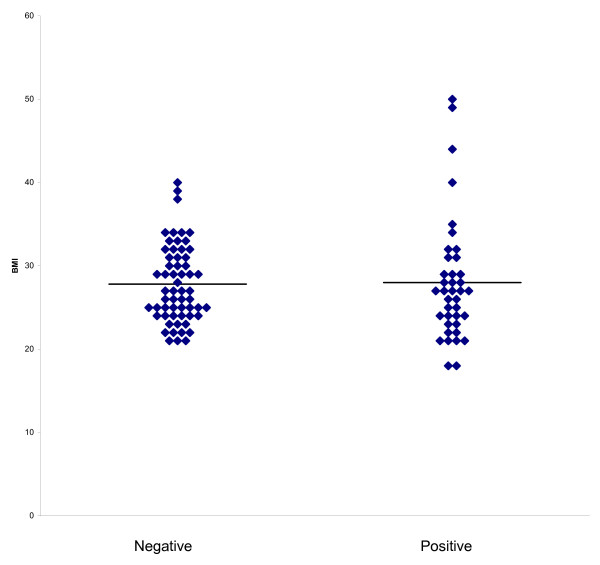
Body mass index according to HER II Overexpression. Horizontal lines are mean.

All results are summarised in table 1. Using the BMI categories of less than 25, 25–30, 30–35 and more than 35 kg/m^2 ^there is no significant trend in the proportions who are HER positive or negative within these categories Figure 1. There was also no association between oestrogen or progesterone receptor positivity and obesity or HER II over expression in premenopausal or post-menopausal women with breast cancer, Figure 1 and Table 1.

**Table 1:** The association between obesity, hormone receptor status and HER II overexpression

	**BMI < 35**	**BMI > 35**	**P value**	**BMI < 30**	**BMI > 30**	**P value**
**HER**	33/88 (38%)	4/6 (67%)	0.21	27/66 (41%)	10/28 (36%)	0.82
**ER**	53/85 (62%)	4/6 (67%)	1	38/65 (58%)	19/26 (73%)	0.24
**PgR**	43/74 (58%)	1/2 (50%)	1	33/58 (57%)	11/18 (61%)	0.79

## Discussion

Obesity is associated with poor prognosis of breast cancer. The exact mechanism by which obesity affects the outcome of breast cancer is not fully understood. HER II overexpression is associated with poor prognosis of breast caner. The present study demonstrated that the poor outcome of breast cancer in obese patients was not due to overexpression of HER II. The present study is in concordance with another small-scale study that showed no association obesity and erbB-2 expression without the use of Fluorescence in-situ hybridisation (FISH) [16]. The present study confirmed that even after FISH analysis there seem to be no association between obesity and HER II expression. These are small-scale studies, and warrant larger scale research.

The relationship between obesity and steroid hormone receptors is complex and controversial. Several relationships have been reported and refuted. Our study is in concordance with other reports, which show no reliable association between steroid hormone receptor and obesity in breast cancer patients.

## References

[B1] Senie RT, Rosen PP, Rhodes P, Lesser ML, Kinne DW (1992). Obesity at Diagnosis of Breast Carcinoma Influences Duration of Disease-Free Survival. Ann Intern Med.

[B2] Petrelli JM, Calle EE, Rodriguez C, Thun MJ (2002). Body Mass Index, Height, and Postmenopausal Breast Cancer Mortality in a Prospective Cohort of US Women. Cancer Causes Control.

[B3] Berclaz G, Li S, Price KN, Coates AS, Castiglione-Gertsch M, Rudenstam CM, Holmberg SB, Lindtner J, Erien D, Collins J, Snyder R, Thurlimann B, Fey MF, Mendiola C, Werner ID, Simoncini E, Crivellari D, Gelber RD, Goldhirsch A (2004). Body Mass Index As a Prognostic Feature in Operable Breast Cancer: the International Breast Cancer Study Group Experience. Ann Oncol.

[B4] Galanis DJ, Kolonel LN, Lee J, Le Marchand L (1998). Anthropometric Predictors of Breast Cancer Incidence and Survival in a Multi-Ethnic Cohort of Female Residents of Hawaii, United States. Cancer Causes Control.

[B5] Jain M, Miller AB (1994). Pre-Morbid Body Size and the Prognosis of Women With Breast Cancer. Int J Cancer.

[B6] Kroenke CH, Chen WY, Rosner B, Holmes MD (2005). Weight, Weight Gain, and Survival After Breast Cancer Diagnosis. J Clin Oncol.

[B7] Goodwin PJ, Boyd NF (1990). Body Size and Breast Cancer Prognosis: a Critical Review of the Evidence. Breast Cancer Res Treat.

[B8] Chlebowski RT, Aiello E, McTiernan A (2002). Weight Loss in Breast Cancer Patient Management. J Clin Oncol.

[B9] Enger SM, Greif JM, Polikoff J, Press M (2004). Body Weight Correlates With Mortality in Early-Stage Breast Cancer. Arch Surg.

[B10] Daling JR, Malone KE, Doody DR, Johnson LG, Gralow JR, Porter PL (2001). Relation of Body Mass Index to Tumor Markers and Survival Among Young Women With Invasive Ductal Breast Carcinoma. Cancer.

[B11] Miller KD, Sledge GW (1999). Toward Checkmate: Biology and Breast Cancer Therapy for the New Millennium. Invest New Drugs.

[B12] Menendez JA, Vellon L, Lupu R (2006). The Antiobesity Drug Orlistat Induces Cytotoxic Effects, Suppresses Her-2/Neu (ErbB-2) Oncogene Overexpression, and Synergistically Interacts With Trastuzumab (Herceptin) in Chemoresistant Ovarian Cancer Cells. Int J Gynecol Cancer.

[B13] Menendez JA, Vellon L, Lupu R (2005). Antitumoral Actions of the Anti-Obesity Drug Orlistat (XenicalTM) in Breast Cancer Cells: Blockade of Cell Cycle Progression, Promotion of Apoptotic Cell Death and PEA3-Mediated Transcriptional Repression of Her2/Neu (ErbB-2) Oncogene. Ann Oncol.

[B14] Colomer R, Menendez JA (2006). Mediterranean Diet, Olive Oil and Cancer. Clin Transl Oncol.

[B15] Anim JT, John B, Abdulsathar SSA, Prasad A, Saji T, Akhtar N, Ali V, Al Saleh M (2005). Relationship Between the Expression of Various Markers and Prognostic Factors in Breast Cancer. Acta Histochem.

[B16] Honda H, Ohi Y, Umekita Y, Takasaki T, Kuriwaki K, Ohyabu I, Yoshioka T, Yoshida A, Taguchi S, Ninomiya K, Akiba S, Nomura S, Sagara Y, Yoshida H (1999). Obesity Affects Expression of Progesterone Receptors and Node Metastasis of Mammary Carcinomas in Postmenopausal Women Without a Family History. Pathol Int.

